# Nano-Encapsulation of Mithramycin in Transfersomes and Polymeric Micelles for the Treatment of Sarcomas

**DOI:** 10.3390/jcm10071358

**Published:** 2021-03-25

**Authors:** Óscar Estupiñán, Claudia Rendueles, Paula Suárez, Verónica Rey, Dzohara Murillo, Francisco Morís, Gemma Gutiérrez, María del Carmen Blanco-López, María Matos, René Rodríguez

**Affiliations:** 1 Instituto de Investigación Sanitaria del Principado de Asturias (ISPA)—Hospital Universitario Central de Asturias, 33011 Oviedo, Spain; o_r_e_s_@hotmail.com (Ó.E.); reyvazquezvero@gmail.com (V.R.); UO278873@uniovi.es (D.M.); 2 Instituto Universitario de Oncología del Principado de Asturias, 33006 Oviedo, Spain; 3 CIBER en Oncología (CIBERONC), 28029 Madrid, Spain; 4 Department of Chemical and Environmental Engineering, University of Oviedo, 33006 Oviedo, Spain; UO251642@uniovi.es (C.R.); UO251076@uniovi.es (P.S.); gutierrezgemma@uniovi.es (G.G.); 5 EntreChem SL, 33011 Oviedo, Spain; fmv@entrechem.com; 6 Asturias University Institute of Biotechnology, University of Oviedo, 33006 Oviedo, Spain; cblanco@uniovi.es; 7 Department of Physical and Analytical Chemistry, University of Oviedo, 33006 Oviedo, Spain

**Keywords:** mithramycin, sarcoma, liposarcoma, chondrosarcoma, encapsulation, transfersomes, nanovesicles, PLGA, micelles

## Abstract

Sarcomas are aggressive tumors which often show a poor response to current treatments. As a promising therapeutic alternative, we focused on mithramycin (MTM), a natural antibiotic with a promising anti-tumor activity but also a relevant systemic toxicity. Therefore, the encapsulation of MTM in nano-delivery systems may represent a way to increase its therapeutic window. Here, we designed novel transfersomes and PLGA polymeric micelles by combining different membrane components (phosphatidylcholine, Span 60, Tween 20 and cholesterol) to optimize the nanoparticle size, polydispersity index (PDI) and encapsulation efficiency (EE). Using both thin film hydration and the ethanol injection methods we obtained MTM-loaded transferosomes displaying an optimal hydrodynamic diameter of 100–130 nm and EE values higher than 50%. Additionally, we used the emulsion/solvent evaporation method to synthesize polymeric micelles with a mean size of 228 nm and a narrow PDI, capable of encapsulating MTM with EE values up to 87%. These MTM nano-delivery systems mimicked the potent anti-tumor activity of free MTM, both in adherent and cancer stem cell-enriched tumorsphere cultures of myxoid liposarcoma and chondrosarcoma models. Similarly to free MTM, nanocarrier-delivered MTM efficiently inhibits the signaling mediated by the pro-oncogenic factor SP1. In summary, we provide new formulations for the efficient encapsulation of MTM which may constitute a safer delivering alternative to be explored in future clinical uses.

## 1. Introduction

Sarcomas comprise a heterogeneous group of malignant neoplasms that arise from mesenchymal stem/progenitor cells (MSCs) and therefore affect mesodermal tissues such as bones, muscles, cartilage or fat. Although these tumors involve only 1% of global cancer diagnoses, this value rises to 12%–15% in terms of pediatric malignancies [1]. In general terms, the treatment of localized sarcomas involves the surgical resection of tumor mass supported by radiotherapy or neoadjuvant chemotherapy [2,3]. In the case of advanced disease or if surgery is not feasible, current treatments still rely on protocols using different cytotoxic drugs. As a result, the overall survival of these patients has remained unchanged for the last 30 years and below 20% in the case of metastatic osteosarcomas and soft tissue sarcomas [2,3], thus highlighting the need for more efficient therapies.

In recent years, several compounds with anti-tumor activity in sarcomas have been reported. Among them, mithramycin A (MTM), also known as Plicamycin, is a natural aureolic acid-type polyketide that was originally isolated from Streptomyces agrillaceus [4]. MTM is able to bind to the DNA minor groove in areas with high GC density, thus inhibiting pivotal transcription factors, such as SP1 and SP3, known to control key oncogenic pathways in cancer [5]. MTM has been used in the past as a therapeutic agent to treat several cancer types, including Ewing sarcoma, in which MTM was able to inhibit its characteristic EWS-FLI1 transcription factor [6]. However, despite its promising efficacy, MTM’s severe side effects have precluded its clinical use at doses which are required to achieve therapeutic responses [7,8]. Nevertheless, new findings describing specific mechanisms of action and drug combinations with synergistic activity in different types of tumors have triggered renewed interest in MTM [6,9,10,11]. In addition, the anti-tumor effects of MTM and related compounds also include strong potential to inhibit the stemness properties in several types of tumors [12,13,14,15,16,17].

Drug delivery using nanocarriers is an interesting option to improve the therapeutic, pharmacological and safety properties of anti-tumor drugs [18]. Thus, the use of nanocarrier systems offers a wide range of possibilities to overcome the multiple physiological barriers that anti-tumor agents must cross, as well as to increase their absorption, distribution and stability in the blood [19,20]. The preferential accumulation of drug-loaded nanocarriers in the tumor is favored by the EPR (enhanced permeability and retention) effect [21], and it may also be actively achieved through the functionalization of the nanoparticles with peptides, aptamers or antibodies, to recognize specific structures in cancer cells [22]. Therefore, some therapeutic nanoparticles (e.g., Doxil^®^, nal-IRI) have received clinical approval for cancer treatment, including sarcomas [23,24].

The use of nanocarriers is especially relevant for drugs like MTM, which show a strong anti-tumoral activity, but a narrow therapeutic window. However, MTM is highly hydrophilic, which makes it more difficult to entrap it using nanocolloids. MTM has been encapsulated before using poly (lactic-co-glycolic acid) (PLGA) [25,26] and other types of polymeric micelles [27] and liposomes [28] as nanocarriers. Interestingly, MTM–PGLA nanoparticles demonstrated a higher anti-tumor activity than free MTM in pancreatic cancer models [26]. PLGA nanoparticles are composed of innocuous and biodegradable polymers formed from co-polymerization of glycolic acid and lactic acid [20]. In addition to polymeric nanoparticles, liposomes and their derivatives are also commonly used for the encapsulation of drugs [29]. Among them, transfersomes are especially flexible and deformable nanovesicles (NVs) with membranes containing a combination of phospholipids (membrane stability), and single chain surfactants (membrane flexibility) [30]. In this work, we designed novel transfersomes and PLGA polymeric micelles able to encapsulate MTM with high efficiency. These drug-delivery systems displayed an optimal average hydrodynamic diameter of 100–200 nm and narrow polydispersity index. In addition, we confirmed the anti-tumor potential of encapsulated MTM in different sarcoma models.

## 2. Materials and Methods

### 2.1. Materials

Phosphatidylcholine (PC) (predominant species: C42H80NO8P, MW= 775.04 g/mol) from soybean (Phospholipon 90G) was a kind gift from Lipoid (Germany). Poly (lactic-co-glycolic acid) (PLGA) (LG 50:50, Mw 24–38 kDa), Sorbitan monostearate (Span 60, S60) (C24H46O6, MW = 430.62 g/mol), Polisorbate 20 (Tween^®^20, T20) (C58H114O26, MM = 1227 g/mol) and Cholesterol (Cho) (C27H46O, MW = 386.65 g/mol) were purchased from Sigma Aldrich (USA). All membrane components were dissolved in absolute ethanol (Sigma Aldrich, St Louis, MO, USA). For the encapsulation experiments 1,2-dioleoyl-sn-glycero-3-phosphocholine-N-(Cyanine 5) supplied by Avanti Polar Lipids, Inc. (Alabaster, AL, USA) was used as fluorescent marker in order to observe under confocal microscopy the nanocolloids and cell interactions. Methanol, acetonitrile, 2-propanol, and the acetic acid of HPLC-grade were supplied by Sigma-Aldrich (USA). A sterile phosphate buffer saline solution (PBS) was used in all experiments as the aqueous phase.

### 2.2. Synthesis of PLGA Polymeric Micelles

In the first set of experiments, several formulations were prepared in order to achieve the proper size, polydispersity index (PDI) and encapsulation efficiency (EE) required to later perform cell tests. These polymeric micelles were prepared by the emulsion solvent evaporation method. An organic phase was prepared with 12.5% (*v*/*v*) methanol in chloroform. PLGA (10 mg/mL) was dissolved in this organic phase. The aqueous phase was prepared with polyvinyl alcohol 1% (*w*/*v*) and membrane compounds. These compounds were S60, PC and Cho, using different molar ratios (for a total of 8.7 mg in 25 mL).

For encapsulation studies, MTM was added on the organic phase. Two milliliters (2 mL) of organic phase were mixed with 6 mL of the aqueous phase and this was sonicated at 2 min (50%) until an emulsion was formed. This emulsion was left under stirring overnight to eliminate the organic phase.

### 2.3. Synthesis of Transfersomes

For the optimization of the size, PDI and EE of these NVs, four different formulations were studied and two different methods of synthesis were used: thin film hydration (TFH) and ethanol injection method (EIM).

Thin film hydration (TFH): Transfersomes were prepared by weighing and dissolving in absolute ethanol PC, S60, T20 and Cho in 1:0.5:0.5:0.5 (TFS1); 1:1.5:1.5:1 (TFS2); 3:3:3:1 (TFS3) and 1.5:1:0.5:1 (TFS4) weight ratios to a concentration of 40 g/L. Then, 100 μL of the organic solution was added to a test tube and the organic solvent was removed using a flow of nitrogen to form a lipid film. The film was rehydrated by adding 2 mL PBS solution, so the final concentration of membrane components was 2 g/L. The resulting vesicular systems were further sonicated for 2 min using an amplitude of 55%.

Ethanol injection method (EIM): Transfersomes were prepared by dissolving the appropriate amount of PC, S60, T20 and Cho in 5 mL of absolute ethanol, for a concentration of 20 g/L. The organic phase was injected, with a syringe pump (KD Scientific, Holliston, MA) at a flow rate of 120 mL/h, into Milli-Q water that was kept at 60 °C and stirred at 500 rpm, so the final concentration of membrane components in aqueous phase was 2 g/L. Then, the ethanol was removed at 40 °C under low pressure (65 mbar) using a rotary vapor. Finally, the vesicular systems were sonicated for 2 min at 55% of amplitude.

For MTM-loaded transfersomes, the same procedure was applied and MTM was added to the organic solution to obtain a final concentration of 1 mg/mL in the aqueous phase (PBS).

### 2.4. Nanocolloids Characterization

#### 2.4.1. Nanocolloids Size

Mean Z-average size and PDI of vesicles were determined via Dynamic Light Scattering (DSL) using a Zetasizer Nano ZS (Malvern Instruments Ltd., UK). Three independent samples were taken from each formulation, and measurements were carried out three times at room temperature without dilution.

#### 2.4.2. Nanocolloids Morphology

The morphological analysis of nanocolloids was carried out by negative staining transmission electron microscopy (NS-TEM), using a JEOL-2000 Ex II TEM (Japan). A sample drop was placed on a carbon-coated copper grid and the sample excess was removed using filter paper.

For the NVs’ characterization, a drop of 2% (*w*/*v*) phosphotungstic acid solution (PTA) was applied to the carbon grid and left to stand for 1 min. Once the excess staining agent was removed with filter paper, the sample was air-dried and the thin film of stained and fixed NVs was observed with the transmission electron microscope.

#### 2.4.3. Encapsulation Efficiency (EE)

EE also gives useful information related to the stability of the vesicle membrane. Hydrophilic compounds are commonly entrapped in the aqueous core surrounded by the lipidic bilayer, while lipophilic components are preferentially located within the surfactant or lipid bilayer [31]. MTM was analyzed by reverse-phase high-performance liquid chromatography (RP-HPLC) (HP series 1100 chromatograph, Hewlett Packard, USA). Before performing these analyses, non-encapsulated MTM was removed by using centrifugal filter units (EMD MilliporeTM AmiconTM Ultra-0.5 centrifugal filter units). Centrifugation was carried out for 20 min at 14600 rpm. Nanocolloids retained in the filter were resuspended in 500 μL sterile PBS solution.

The RP-HPLC system was equipped with a UV/VIS absorbance detector HP G1315A and a fluorescence detector 1260 Infinity A (Agilent Technologies, Santa Clara, CA, USA). The column was a Zorbax Eclipse Plus C18 of 5 µm particle size, 4.6 mm × 150 mm (Agilent Technologies, USA). The mobile phase consisted of a mixture of (A) 100% milliQ-water; and (B) 100% methanol with gradient elution at 0.8 mL/min. The step gradient started with a mobile phase of 80%: (A) running 100% mobile phase; (B) in minute 5 for 10 min. The mobile phase (B) was fed for 2 min after each injection to prepare the column for the next sample. The analysis was carried out at 30 °C. The wavelength used for UV/VIS detector was 280 nm.

### 2.5. Cell Culture, Drugs and Ethics Statement

The myxoid liposarcoma model cell line MSC-5H-FC was generated by the sequential transformation of human bone marrow-derived mesenchymal stem/stromal cells (hBM-MSCs) with up to six oncogenic hits, including the fusion oncogene FUS-CHOP (FC), as previously reported and characterized [32,33]. The related cell line T-5H-FC#1 was derived from a xenograft tumor generated by MSC-5H-FC cells [33,34]. T-CDS17#4 is a cell line derived from a xenograft generated by the chrondrosarcoma primary cell line CDS17, which were fully described in a preceding work [35]. Healthy human BM-MSCs were obtained from Inbiobank (San Sebastian, Spain) and were functionally and phenotypically characterized in a previous work [32]. The identity of these cell lines has been authenticated by a short tandem repeats analysis during the last 5 months. All the cell lines were tested monthly for mycoplasma using the LONZA MycoAlert Mycoplasma Detection Kit (LONZA, Rockland CE) and cultured as previously described [33,35]. MTM was synthesized by EntreChem SL (Oviedo, Spain) as previously described [36]. Stocks of MTM were prepared as 10 mM solutions in DMSO, maintained at −20 °C, and diluted in culture medium to the final concentration just before use. All experimental protocols have been performed in accordance with institutional review board guidelines and were approved by the Institutional Ethics Committee of the Principado de Asturias (ref. 255/19).

### 2.6. Cell Viability Assays

Cell viability of cell lines after the treatment with increasing concentrations of free MTM or MTM encapsulated in different nanocarriers was assayed using the Cell Proliferation reagent WST-1 (Roche, Mannheim, Germany) as described before [37]. The concentration of the half-maximal inhibition of viability (IC50) for each condition was calculated by nonlinear regression using the GraphPad Prism software (La Jolla, CA, USA).

### 2.7. Tumorsphere Culture

Cells were plated at a density of 5000 cells per well in 6-well low-attachment plates (Sigma, St Louis, MO, USA) and cultured in serum-free sphere medium containing DMEM-F12 (GE Healthcare, Pittsburg, PA, USA) supplemented with Glutamax (1:100; Life Technologies, Carlsbad, CA, USA), B-27 Supplement (1:50; Life Technologies), Heparin (1:1000; Sigma), the growth factors human EGF (20 ng/mL) and human bFGF (10 ng/ ml; PeproTech, London, UK). Fresh aliquots of EGF and bFGF were added every three days. To analyze the effects of drugs, the tumorspheres formed after 10 days of culture were incubated in sphere medium containing different concentrations of the drugs for 3 days. After the treatments, tumorspheres were scored and cell viability was determined using the Cell Proliferation reagent WST-1. [38].

### 2.8. Western Blotting

The preparation of protein extracts and the protocol for Western blotting analysis were previously reported [39]. The following primary antibodies were used at the indicated dilutions: anti-SP1 [(9389), 1:1000 dilution] from Cell Signaling (Danvers, MA, USA); anti-c-MYC [(sc-40), 1:100], anti-VEGF [(sc-57496), 1:100], and anti-IGF1-R [(sc-81464, 1:100] from Santa Cruz Biotechnology (Dallas, TX, USA); and anti-β-Actin [(A5441), 1:10,000] from Sigma. IRDye Infrared Fluorescent secondary antibodies IRDye 800CW and IRDye-680RD (LI-COR Biosciences, Lincoln, NE, USA) were used for signal detection using an Odyssey Fc imaging system and the software Image Studio (LICOR). Uncropped images of the Western blots are shown in Figure S1.

### 2.9. Nanocolloids/MTM Confocal Imaging

Synthesized nanocolloids were tagged by adding 1% (*v*/*v*) of headgroup Cy5 labelled PC (850483C, Avanti Polar Lipids, Alabaster, Alabama, USA) to the membrane component mixture. After an in vitro treatment, MTM and labelled PLGA polymeric micelles were observed by confocal microscopy using Leica TCS-SP8X microscope (Photon Microscopy and Image Processing Unit, University of Oviedo). MTM detection was performed at 395/535 nm excitation/emission pair and Cy5 tagged nanoparticles at 649/666 nm.

## 3. Results

### 3.1. Nanocolloids Characterization

**Figure 1:**
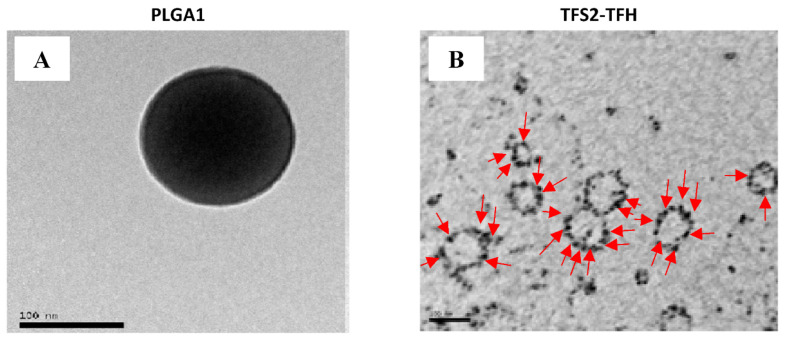
TEM micrographs of PLGA polymeric micelles and transfersomes containing mithramycin (MTM): (**A**) PLGA polymeric micelles prepared with Span60:Cholesterol (1:0.5)(PLGA1) by the solvent evaporation method containing MTM; (**B**) transfersomes formulated with PC:S60:20:Cho (1:1.5:1.5:1) prepared by the TFH method (TFS2-TFH) containing MTM. Red arrows indicate the presence of MTM in the membrane bilayer of transfersomes. Scale bar: 100 nm.

Table 1 summarizes mean sizes, PDI and EE values of both PLGA polymeric micelles and transferosome NVs. The sizes obtained by DLS were in the range of 210–267 nm for the PLGA polymeric micelles, which is an indication that all formulations present an appropriate size with a narrow PDI between 0.083 and 0.137. It is important to point out that the size measured by DLS corresponds to the hydrodynamic diameter which is normally larger than the one observed under TEM due to the double layer of ions and counterions that surround the nanocolloids in solution. TEM micrographs (Figure 1A) revealed sizes in the range of 100–150 nm. It was also observed that EE was higher in PLGA1 (S60:Cho) polymeric micelles (87%), formulated using cholesterol and without PC. This is in line with previous studies showing that cholesterol at low concentrations can increase EE [40].

Transfersomes include the presence of both phospholipids and surfactants, sharing the properties of both types of systems. For the TFS formulations, the amount of membrane compounds added to the organic phase was optimized using PC, Span^®^60, Tween^®^20 and Cho membrane components. This formulation was used since it is one of the most common formulations studied in previous works [41,42,43]. Cho is commonly used as a membrane stabilizer for NV preparation to improve stability, membrane elasticity and EE. However, cholesterol slightly increases vesicle size. It has also been reported that Cho plays an important role in niosome formulation when hydrophilic surfactants (as is the case of Tween^®^20) are used as the main membrane compounds [41]. The sizes obtained by DLS were in the range of 97–133 nm, with similar sizes observed in TFS–TFH and TFS–EIM nanoparticles. PDI values were relatively low for TFS–EIM formulations while TFS–TFH particles showed a broader size distribution (PDI values between 0.37 and 0.40), which is consistent with the literature when this method is used [44]. TEM micrographs were in good agreement with the sizes reported by DLS for all TFS formulations (Figure 1B). EE values were similar for both methods of transferosome preparation and varied from 50% to 58%. Interestingly, MTM accumulated in the membrane bilayer of the TFS2–TFH nanoparticles, thus indicating that MTM has been entrapped in the lipophilic region of these NVs. (Figure 1B). This finding was in good agreement with the previous results reporting that hydrophilic molecules could interact with the hydrophilic portion of the membrane compounds at the NVs/water interface of encapsulating NVs when they were prepared by the TFH method [44].

For the subsequent testing of the cytotoxic activity of encapsulated MTM in sarcoma cells, we chose PLGA1 formulation, since it represents the polymeric micelles with the highest EE percentage and an adequate size. Regarding transferosome particles, given the similarity of sizes and EE obtained by both methods, TFS2-TFH and TFS4-TFH formulations were selected for further testing due to the great advantage this method offers, since it allows the preparation of a rather smaller volume of samples compared to the EIM one. The MTM concentration achieved in nanoparticles ranged from 443 to 547 µg/mL for PLGA1, from 365 to 463 µg/mL for TFS2–TFH, and from 433 to 579 µg/mL for TFS4–TFH. These concentrations allow the treatment of pre-clinical models with doses above the maximum tolerated dose described for MTM (2 mg/Kg).

### 3.2. Free and Encapsulated MTM Shows Similar Cytotoxic Effect in Sarcoma Cells

**Figure 2:**
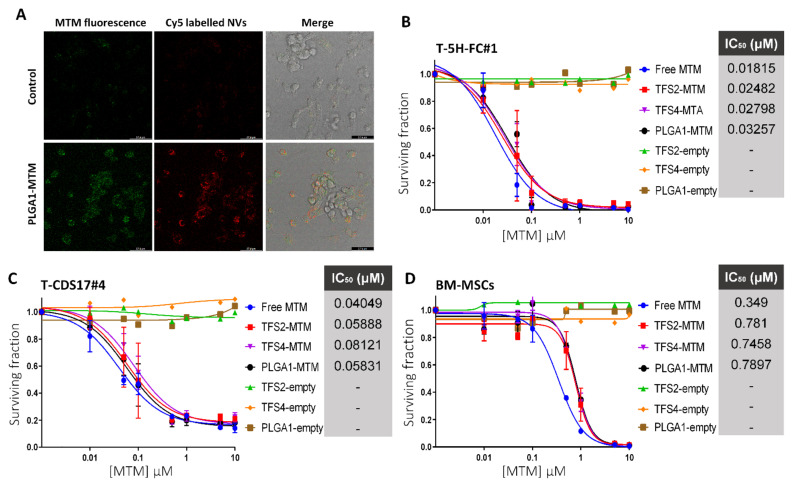
Anti-proliferative effect of the free MTM and nano-encapsulated MTM in sarcoma cells and hBM-MSCs: (**A**) confocal microscopy detection of fluorescence emitted by MTM (green fluorescence; left panels) and Cy5 (red fluorescence; middle panels) in T-5H-FC#1 cells treated with DMSO (control) or 1 µM MTM loaded in Cy5-labelled NVs-C for 2 h. Bright field images merged with green and red fluorescence emissions are also shown (right panels). Bar scale = 57.9 µm. (B–D) Cell viability (WST1 assay) measured after the treatment of the mixoid liposarcoma model T-5H-FC#1 (**B**); the patient-derived chondrosarcoma cell line T-CDS17#4; (**C**) or a culture of non-transformed hBM-MSCs (**D**) with increasing concentrations of MTM for 72 h. The effect of equivalent amounts of empty nanoparticles are shown. IC50 values for each treatment are indicated. Error bars represent the standard deviation of three independent experiments.

To study the ability of encapsulated MTM to target sarcoma cells, we first prepared MTM-loaded PLGA1 polymeric micelles including the headgroup Cy5 labelled PC and detected both MTM- (535 nm) and Cy5-emitted fluorescence (666 nm) by confocal microscopy. After the two-hour treatment of the liposarcoma model, T-5H-FC#1 cells with MTM-loaded PLGA nanoparticles we observed both types of fluorescence in most cells, thus indicating that nanoparticles can efficiently enter and release MTM inside sarcoma cells (Figure 2A).

Then, we compared the cytotoxic effect induced by free MTM and MTM loaded in PLGA1 (PLGA1–MTM), TFS2–TFH (TFS2–MTM), and TFS4–TFH (TFS4–MTM) nanoparticles in sarcomas cells. In these experiments, we found that MTM encapsulation does not affect its anti-tumor activity. Thus, T-5H-FC#1 liposarcoma cells (IC50 ≈ 18–32 nM) and T-CDS17#4 chondrosarcoma cells (IC50 ≈ 40–81 nM) were both sensitive to the nanomolar concentrations of all assayed MTM formulations (Figure 2B,C).

The anti-proliferative activity of free MTM in the primary cultures of non-transformed human BM-MSCs (IC50 = 350 nM) is 10–20 lower than that observed in T-5H-FC#1 cells, originally derived from transformed BM-MSCs. Interestingly, MTM encapsulation in any of the assayed nano-formulations was able to reduce two times the anti-proliferative activity of free MTM in healthy BM-MSCs (IC50 ≈ 745–790 nM) (Figure 2D). In all cases, empty nanoparticles do not show any cytotoxic effect in both neoplastic or healthy cells at equivalent concentrations of MTM-loaded nanoparticles (Figure 2B–D).

### 3.3. Nano-Encapsulated MTM Targets Cancer Stem Cells (CSCs) Subpopulations in Sarcoma.

Cancer stem cell (CSC) subpopulations are thought to be responsible for tumor maintenance, being directly associated with invasion and metastatic dissemination [45]. Relevantly, MTM has demonstrated its potential to eliminate CSC subpopulations in different types of cancer [12,14,15,17]. CSC-enriched cell culture can be obtained by growing cells as floating 3D tumorspheres [34]. Therefore, we tested the ability of the different MTM formulations to target CSCs by analyzing their anti-proliferative effect in tumorsphere cultures.

**Figure 3:**
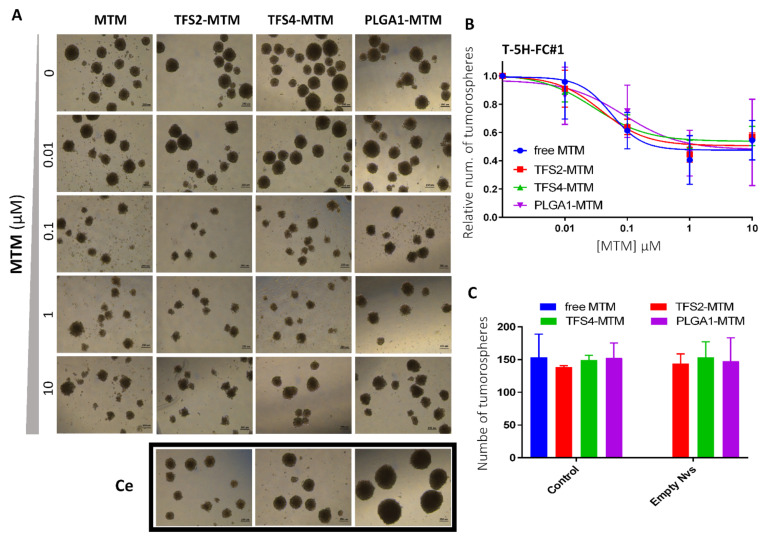
Effect of free and nano-encapsulated MTM in CSC-enriched 3D sarcoma cell cultures. (**A**) Cells were plated at low density in tumorsphere medium and left to form tumorspheres for 10 days before treating them for 72 h with increasing concentrations of MTM. Treatments with an amount of empty nanoparticles corresponding to 10 µM (Ce) were also included. Scale bars = 250 μm. (**B**,**C**) Quantification of the spheres (represented as % of control) remaining after the treatment with MTM-loaded nanoparticles; (**B**) or with empty nanoparticles (**C**). Error bars represent the standard deviation of three independent experiments.

Using this 3D model, we find that both free MTM and all assayed MTM nano-delivery systems are equally able to decrease the number and the size of T-5H-FC#1 tumorspheres (Figure 3A,B). Furthermore, empty nanoparticles do not induce any cytotoxic effect against CSC-enriched cultures (Figure 3C).

### 3.4. Free and MTM-Loaded Nanoparticles Inhibit SP1 Pathway Similarly

**Figure 4:**
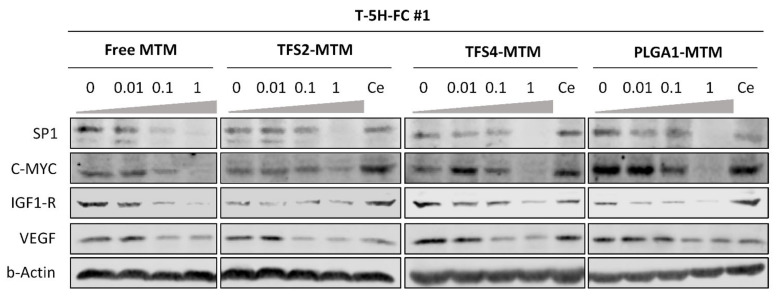
Inhibition of SP1 signaling by free and nano-encapsulated MTM. Western blotting analyses of SP1 and several SP1 downstream targets in T-5H-FC#1 cells treated with the indicated concentrations of the different MTM formulations for 24 h. A treatment with empty nanoparticles (Ce) at a concentration equivalent to the higher encapsulated MTM dosage was also included. The expression of β-actin was used as the loading control.

The anti-tumor activity of MTM relies on its capacity to bind GC-rich DNA regions and thus inhibit the gene expression mediated by pro-oncogenic transcription factors such as SP1 [5,46,47]. Therefore, we characterized the ability of the different MTM formulations to repress at the protein level the expression of SP1 and a panel SP1 downstream targets, such as C-MYC, IGF1-R and VEGF [48]. We find that both free and encapsulated MTM induce an efficient dose–response-dependent repression of all analyzed targets in T-5H-FC#1 cells (Figure 4). An MTM concentration of 1 µM was able to almost completely inhibit the expression of all targets after 24 h of treatment. Moreover, no effect was observed after the treatment with empty nanoparticles (Figure 4).

These results confirmed that MTM encapsulated in polymeric nanoparticles and transfersomes mimic the ability of free MTM to inhibit the expression of important pro-tumor factors in sarcoma cells.

## 4. Discussion

MTM is a natural antibiotic approved by the FDA for the treatment of hypercalcemia [49]. This drug was also evaluated in the past for the treatment of several leukemias and solid tumors. Indeed, MTM demonstrated good anti-tumor responses in patients with testicular carcinoma, glioblastoma or Ewing sarcoma [6,50,51]. However, the appearance of severe systemic toxicities, in combination with the development of other successful and less toxic treatment regimens has limited the clinical use of MTM [7,8,50,52]. More recently, the discovery of new findings regarding the ability of MTM to block the signaling mediated by pro-oncogenic factors, such as SP1, EWS-FLI1, or SMARCB1-deficient SWI/SNF chromatin remodeling complexes, has renewed interest in this drug [5,6,9,46,47,53].

To clinically exploit these interesting anti-tumor properties of mithramycin while managing its adverse effects, different strategies have been developed. First, the development of second-generation mithramycin analogs, such as EC-8042, which show a similar efficacy in inhibiting SP1-mediated transcription activity and anti-stemness potential but much less toxicity, open the possibility of using these compounds in the clinic [16,54]. Additionally, according to newly described mechanisms of action of MTM, drug combinations with synergistic activity in different tumor types have been reported. Thus, recent studies have found that the combination of MTM with CDK9 inhibitors or the semisynthetic taxane cabazitaxel resulted in improved anti-tumor activity and/or limited MTM-toxicity in Ewing sarcoma [10] and B-cell acute lymphoblastic leukemia [11], respectively. Finally, MTM encapsulation in different delivery systems is being evaluated as a way to improve its clinical utility. A few studies previously reported protocols to encapsulate MTM in Ca^2+^-loaded large unilamellar vesicles (LUVs) [28], in PLGA nanoparticles through single-emulsion solvent evaporation or nanoprecipitation techniques [25,26], and other types of polymeric micelles using microfluidic technology [27]. In addition, several MTM analogues have also been formulated in cross-linked micelles [55]. Here, we used new refined methods to synthesize controlled size PLGA polymeric micelles and transfersomes that allow the direct encapsulation of MTM. For the synthesis of PLGA micelles, we tested different nanoparticle formulations by modifying the main amphiphilic component of the micelle, i.e., using both non-ionic surfactant, phospholipid, or a combination of both. In addition, we also studied the effect of adding a membrane stabilizer (cholesterol) following the method (emulsion/solvent evaporation) reported by Wang et al. to encapsulate doxorubicin [56]. This could affect the interactions between the polar heads or hydrophobic chains with the PLGA and MTM and therefore affect the final colloidal stability, size, PDI and EE. The ability of our PLGA micelles to entrap MTM (up to 87%) were higher than that reported by Wang et al. for doxorubicin encapsulation (44%) using the same method with soybean lecithin as the main micelle component [56]. This method was also used by Liu et al. to encapsulate MTM in poly-ethylene glycol-coated PLGA nanoparticles [26]. The formulations used by these authors yielded smaller nanoparticles (25 nm) with a lower EE of 29.5%. In addition, MTM was encapsulated in by Cohen-Sela et al. in PLGA nanoparticles using different approaches. These authors show that a modified emulsion/solvent method formed nanoparticles of 110 nm with EE values of 41%, while a double emulsion solvent diffusion method or a nanoprecipitation technique produced particles of 130–160 nm and a higher EE of 80%, similar to that measured in our PLGA micelles [25]. Capretto et al. demonstrated that MTM can be efficiently encapsulated in polymeric micelles based on Pluronic^®^ block copolymers by a new microfluidic approach. In this case, the sizes obtained were smaller, as expected regarding the microfluidic method used, being in the range of 60–90 nm, with a percentage of drug incorporated in the polymeric micelles ranging from 55% to 92% but with a total concentration of MTM encapsulated from 0.5 to 40 µg/mL [27].

Similar to polymeric nanoparticles, the size of lipidic NVs is also governed by their composition and preparation method. Previous studies by our group aimed at optimizing the encapsulation of compounds of various natures in different types of NVs (niosomes, liposomes and transfersomes) showed that both EIM and TFH methods were capable of producing transfersomes with a favorable size range (100–200 nm) and high EE values [41,57]. Notably, TFH has demonstrated its potential to efficiently entrap difficult-to-encapsulate hydrophilic drugs, as is the case with MTM [44]. The only previous attempt to encapsulate MTM in lipid nanoparticles was reported in 1997 by Frézard et al. These authors showed that MTM can be indirectly encapsulated within LUVs, made from dipalmitoylphosphatidylcholine and cholesterol, only when the liposomes containing Ca^2+^ and were resuspended in a Ca^2+^-free medium. Using this formulation, MTM and Ca^2+^ formed a high affinity complex inside the inner aqueous core of the liposomes, leading to reach EE values up to 60% [28]. In our study, we produced transfersomes presenting similar EE values and also with the advantage that the preparation methods used here, both EIM and TFH, which allows direct encapsulation, thus facilitating subsequent applications as nanocarriers for cancer cell treatment.

Here, we used our MTM nano-delivery systems to assay its potential to target sarcoma cells. We showed that all assayed MTM nano-formulations retain the efficient anti-tumor activity of free MTM, both in a cell-of-origin model of sarcoma-initiating cells and a patient-derived model of chondrosarcoma. Interestingly, MTM-nanoparticles resulted as less toxic than free MTM to normal BM-MSCs. Therefore, it may be speculated that these nanocarriers have the potential to increase the therapeutic window of MTM. The exact mechanism by which nano-encapsulated MTMs show a safer profile in healthy cells in vitro is yet to be clarified, but it may be due to the differential effect of the sustained drug release in tumoral and non-tumoral cells, as suggested for other drugs such as doxorubicin [58]. In any case, future in vivo analyses should be performed to confirm that MTM encapsulation is able to reduce the systemic toxicity of this drug, making possible the safe administration of concentrations capable of inhibiting tumor growth in vivo.

The cytotoxic effect of nanocarrier-delivered MTM is accompanied by an efficient ability to inhibit the expression of SP1 and other well-known SP1 downstream targets. This transcription factor has been reported to control tumor growth and promote drug resistance both in soft tissue sarcoma [59,60] and bone sarcomas [61]. Relevantly, we previously showed that the inhibition of SP1 with the mithramycin analog EC-8042 was able to efficiently inhibit the growth of our myxoid liposarcoma model [13,16]. Among the SP1 downstream targets, that we found to be efficiently downregulated by MTM, there are some of the genes most commonly amplified/upregulated in sarcomas, such as C-MYC, VEGF or IGF1R [62,63,64,65]. Several targeted therapies against most of these factors have been investigated and used for sarcoma treatment in recent years. For instance, anti-VEGF treatments, such as apatinib or pazopanib, have been used for the treatment of leiomyosarcoma and other soft tissue sarcomas [1,66]. However, some improvements have been found in specific cases, where these targeted therapies directed to a particular target have not demonstrated a higher efficacy as first-line treatments than standard chemotherapy in most cases of sarcoma. We speculate that treatments such as nano-delivered MTM, which are able to simultaneously inhibit several well-known driver oncogenes in sarcoma, may have a higher impact on the patient’s outcome.

Another recently reported interesting property of MTM and MTM analogues is their ability to target the tumorigenic and invasive CSC subpopulations in different tumor types. Thus, MTM was able to downregulate the expression of genes associated with the CSC phenotype and target CSC subpopulations in glioblastoma [15], medulloblastoma [17], lung cancer [12] and colon cancer [14]. In a similar way, we showed that EC-8042 was able to inhibit the expression of a panel of CSC markers and demonstrated a higher ability to target CSCs in vivo than other chemotherapeutics drugs in sarcoma cells [13,16]. Here, we show that nano-encapsulated MTM is able to reduce the viability of CSC-enriched tumorspheres, hence confirming that these formulations keep the anti-stemness potential of free MTM and MTM analogues.

## 5. Conclusions

Overall, we provide new formulations for the efficient nano-encapsulation of MTM. These MTM-delivery systems retain the anti-tumor and anti-stemness potential of free MTM in sarcoma cells, therefore suggesting that they may constitute a safer MTM-delivering alternative to be explored for future clinical uses.

## Figures and Tables

**Table 1:** Polymeric micelles and transfersomes size (nm) in terms of Z average, intensity and number, polydispersity index (PDI), encapsulation efficiency (EE).

	Formulation	Z-Average Size (nm)	PDI	EE (%)
PLGA polymeric micelles	PLGA1 S60:Cho (1:0.5)	216 ± 26	0.101 ± 0.039	87 ± 15
	PLGA2 S60	267 ± 57	0.137 ± 0.02	82.5 ± 4.0
	PLGA3 PC	210 ± 5	0.084 ± 0.02	63.8 ± 12.3
	PLGA4 S60:PC (1:1)	221 ± 2	0.083 ± 0.02	76.4 ± 14.5
TFS–EIM	TFS1–EIM PC:S60:20:Cho (1:0.5:0.5:0.5)	115 ± 9	0.240 ± 0.018	58 ± 15
	TFS2–EIM PC:S60:20:Cho (1:1.5:1.5:1)	126 ± 4	0.246 ± 0.004	52 ± 18
	TFS3–EIM PC:S60:T20:Cho (3:3:3:1)	117 ± 5	0.235 ± 0.021	51 ± 10
	TFS4–EIM PC:S60:T20:Cho (1.5:1:0.5:1)	126 ± 3	0.244 ± 0.005	50 ± 11
TFS–TFH	TFS1–TFH PC:S60:20:Cho (1:0.5:0.5:0.5)	107 ± 4	0.400 ± 0.008	52 ± 14
	TFS2–TFH PC:S60:20:Cho (1:1,5:1,5:1)	127 ± 29	0.382 ± 0.074	50 ± 16
	TFS3–TFH PC:S60:T20:Cho (3:3:3:1)	97 ± 7	0.388 ± 0.053	56 ± 4
	TFS4–TFH PC:S60:20:Cho (1.5:1:0.5:1)	133 ± 39	0.366 ± 0.074	52 ± 11

## Data Availability

Data presented in this study are available in Supplementary Figure S1.

## Supplementary Materials

The following are available online at https://www.mdpi.com/2077-0383/10/7/1358/s1, Figure S1: Whole Western blotting images from Figure 4.
